# NLRP3 Inflammasome Assembly in Neutrophils Is Supported by PAD4 and Promotes NETosis Under Sterile Conditions

**DOI:** 10.3389/fimmu.2021.683803

**Published:** 2021-05-28

**Authors:** Patrick Münzer, Roberto Negro, Shoichi Fukui, Lucas di Meglio, Karen Aymonnier, Long Chu, Deya Cherpokova, Sarah Gutch, Nicoletta Sorvillo, Lai Shi, Venkat Giri Magupalli, Alexander N. R. Weber, Rüdiger E. Scharf, Clare M. Waterman, Hao Wu, Denisa D. Wagner

**Affiliations:** ^1^ Program in Cellular and Molecular Medicine, Boston Children’s Hospital, Boston, MA, United States; ^2^ Department of Pediatrics, Harvard Medical School, Boston, MA, United States; ^3^ Department of Cardiology and Angiology, University of Tübingen, Tübingen, Germany; ^4^ Whitman Center, Marine Biological Laboratory, Woods Hole, MA, United States; ^5^ Department of Biological Chemistry and Molecular Pharmacology, Harvard Medical School, Boston, MA, United States; ^6^ Laboratory of Vascular Translational Science, U1148 INSERM University of Paris, Paris, France; ^7^ Department of Immunology, Interfaculty Institute of Cell Biology, University of Tübingen, Tübingen, Germany; ^8^ Division of Experimental and Clinical Hemostasis, Hemotherapy, and Transfusion Medicine, and Hemophilia Comprehensive Care Center, Institute of Transplantation Diagnostics and Cell Therapy, Heinrich Heine University Medical Center, Düsseldorf, Germany; ^9^ Cell Biology and Physiology Center, National Heart, Lung, and Blood Institute of the National Institutes of Health, Bethesda, MD, United States; ^10^ Division of Hematology/Oncology, Boston Children’s Hospital, Boston, MA, United States

**Keywords:** Neutrophils, NETs, NLRP3 inflammasome, MCC950, deep vein thrombosis, PAD4

## Abstract

Neutrophil extracellular trap formation (NETosis) and the NLR family pyrin domain containing 3 (NLRP3) inflammasome assembly are associated with a similar spectrum of human disorders. While NETosis is known to be regulated by peptidylarginine deiminase 4 (PAD4), the role of the NLRP3 inflammasome in NETosis was not addressed. Here, we establish that under sterile conditions the cannonical NLRP3 inflammasome participates in NETosis. We show apoptosis-associated speck-like protein containing a CARD (ASC) speck assembly and caspase-1 cleavage in stimulated mouse neutrophils without LPS priming. PAD4 was needed for optimal NLRP3 inflammasome assembly by regulating NLRP3 and ASC protein levels post-transcriptionally. Genetic ablation of NLRP3 signaling resulted in impaired NET formation, because NLRP3 supported both nuclear envelope and plasma membrane rupture. Pharmacological inhibition of NLRP3 in either mouse or human neutrophils also diminished NETosis. Finally, NLRP3 deficiency resulted in a lower density of NETs in thrombi produced by a stenosis-induced mouse model of deep vein thrombosis. Altogether, our results indicate a PAD4-dependent formation of the NLRP3 inflammasome in neutrophils and implicate NLRP3 in NETosis under noninfectious conditions *in vitro* and *in vivo*.

## Introduction

Initially described as part of the innate immune response to microbes (1), there is now increasing evidence that neutrophil extracellular traps (NETs) are produced under sterile conditions. They are implicated in a wide variety of inflammatory, (auto)immune, and thrombo-occlusive disorders. In particular, NETs are known to foster thrombosis (2, 3), contribute to ischemia/reperfusion injury (4), and age-related tissue fibrosis (5). NETs also likely contribute to the severe side effects of a COVID-19 infection (6). Moreover, NET formation is stimulated by diseases, such as diabetes (7) and cancer (8), and contributes to cancer progression (9, 10).

NETs are decondensed chromatin meshworks ejected by neutrophils upon inflammatory stimulation or hypoxia. A key characteristic of the extracellular neutrophil chromatin is the inclusion of pro-thrombotic, pro-inflammatory, and cytotoxic components, in particular histones and microbicidal proteases (3, 11). While the clinical relevance of NETs is recognized, the underlying cellular mechanisms of their induction are poorly defined. Recently, NET formation (NETosis) was determined to be a well-orchestrated sequence of cellular events, including disassembly of the cellular cytoskeletons, endomembrane fragmentation, nuclear rounding, plasma membrane permeabilization, and finally nuclear and plasma membrane rupture (12).

A major prerequisite for NETosis is the peptidylarginine deiminase 4 (PAD4)-dependent post-translational modification of histones (13, 14). In general, PAD enzymes are calcium-dependent enzymes which deiminate the positively charged arginine residue of proteins, thus transforming arginine to a neutral citrulline. PAD4 is mainly expressed in granulocytes and transferred into an enzymatic active conformation upon calcium binding. Moreover, since PAD4 is the only PAD isoform that contains a nuclear localization sequence, it is required for nuclear histone citrullination (12, 15). During the course of NETosis, it is thought that citrullination of histones reduces their DNA/histone binding ability which causes chromatin decondensation and subsequently culminates in chromatin expulsion.

Effective inducers of NETosis *in vitro* are the calcium ionophore ionomycin, the protein kinase C activator phorbol 12-myristate 13-acetate (PMA), and the potassium ionophore nigericin (16, 17). Interestingly, nigericin is widely used in macrophages to induce assembly of the NLR family pyrin domain containing 3 (NLRP3) inflammasome, which is also expressed in neutrophils (18, 19). In neutrophils the NLRP3 inflammasome was found to be activated after bacterial infection (20, 21) or after lipopolysaccharide (LPS) pretreatment with subsequent ATP stimulation (22). In addition, an activating mutation (A352V) in NLRP3 leading to Muckle Wells syndrome is associated with excessive neutrophil granule exocytosis (23) and a gain-of-function mutation in NLRP3, which results in Familial Mediterranean Fever (FMF) is subsequently linked to augmented NETosis (24, 25).

Inflammasomes are multiprotein signaling platforms, described mainly in macrophages, that mediate pivotal responses of innate immunity after activation by pathogen- and danger-associated molecular patterns (PAMPs and DAMPs). The most prominent and best studied inflammasome is NLRP3 (26, 27). Assembly of the NLRP3 inflammasome in macrophages *in vitro* requires a two-step mechanism. First, protein expression levels of NLRP3 have to be increased transcriptionally by priming macrophages with lipopolysaccharide (LPS) and, second, subsequent stimulation for example with pore-forming toxins (nigericin), results in a prominent NIMA (never in mitosis gene a)-related kinase 7 (NEK7)-dependent oligomerization of NLRP3 (28, 29). Successively, apoptosis-associated speck-like protein containing a CARD (ASC) is recruited, polymerized, and crosslinked with pro-caspase-1 leading to the formation of a macromolecular multiprotein structure designated ASC speck (30). ASC speck formation results in activation of caspase-1 (31), which in turn allows processing of the pro-inflammatory cytokines IL-1ß and IL-18, as well as the pore-forming protein gasdermin D (GSDMD) (32). Specific inhibition by the small molecule MCC950, an established NLRP3 inhibitor that binds to NLRP3 (33), results in impaired ATP hydrolysis with a subsequent blockade of NLRP3 inflammasome formation (34, 35).

Interestingly, neutrophils also have been described as a source of NLRP3/ASC-dependent IL-1ß production after *Staphylococcus aureus* infection (36). NLRP3-linked disorders, like hypoxia-induced venous thromboembolism (37), atherosclerosis (27), and tissue damage after ischemia/reperfusion (38) have an inflammatory thrombo-occlusive pathology in common that is associated with PAD4 and NETosis. So far, NLRP3 assembly and ASC speck formation have been described in neutrophils only after pathogen-induced infections (19, 20) or in the presence of LPS (22). Nothing is known about NLRP3 inflammasome assembly in neutrophils in sterile inflammation or their potential role in NETosis.

Here, we demonstrate that the formation of the NLRP3 inflammasome supports NETosis in the absence of LPS both *in vitro* and *in vivo*, and that PAD4, in addition to its known role in chromatin decondensation, also regulates NLRP3 inflammasome assembly in neutrophils. Our studies provide an important link between NETosis and the NLRP3 inflammasome, explaining, at least in part, the overlapping features of disorders in which both components are involved.

## Materials and Methods

### Materials

A detailed list of used material and corresponding ordering informations can be found in the **Supplementary Informations**.

### Animals

*Nlrp3^–/–^* (stock no. #021302) and corresponding wild-type (C57BL/6J; stock no. #000664) mice were obtained from Jackson Laboratory (Bar Harbor, ME, USA). *Padi4^–/–^* mice were originally generated by Y. Wang (13) and back-crossed with C57BL/6J in the Wagner laboratory. All mouse lines were housed in the animal facility of Boston Children’s Hospital.

*Padi4^fl/fl^* mice (stock no. #026708), previously described by Hemmers and colleagues (39), and *Vav1-iCre* mice (stock no #008610) were purchased from Jackson Laboratory and intercrossed by the Wagner laboratory to generate mice lacking PAD4, specifically in the hematopoietic lineage (*Padi4^Vav1Cre/+^*). ASC-deficient mice (C57BL/6J background) used for antibody validation were a kind gift of A. Yazdi (Aachen University, Germany) and were previously described (40). All offsprings were housed in the according institutional animal facility, and mice of both sexes were randomly assigned for experiments. Data analysis was blinded to the identity of the sample.

All experimental animal procedures in this study were approved by the Institutional Animal Care and Use Committee of Boston Children’s Hospital under the protocol numbers 20-01-4096R or 20-02-4097R or the Regierungspräsidium Tübingen and were performed under the ARRIVE guidelines.

### Flow Restriction Model (DVT)

Flow restriction of the inferior vena cava (IVC) was performed as described elsewhere (41). Briefly, the IVC of 8-week-old male *Nlrp3^–/–^* and corresponding wild-type mice was exposed, and the renal and iliac veins were ligated. Subsequently, the IVC was partially (90%) ligated with a 7-0 polypropylene suture using a 30-gauge needle as a spacer. After removal of the spacer, the peritoneum and skin were closed by monofilament sutures, and mice were euthanized 6 or 48 hours after surgery. Formed thrombi were harvested for weight and length measurements and cryo-embedded in Tissue-Tek^®^ O.C.T.™.

### Immunofluorescence Staining of Thrombi

Cryo-embedded thrombi were cryo-sectioned into 10 µm sections and fixed in 4% paraformaldehyde (PFA) overnight at 4°C. After being washed once with phosphate-buffered saline (PBS), thrombi sections were permeabilized (0.1% Triton X-100, 0.1% sodium citrate) for 10 minutes at 4°C and subsequently incubated with blocking buffer (2.5% BSA, 0.5% Tween-20 in 1x PBS) at 37°C for 1 hour. Following incubation with the primary antibodies H4Cit (1:250) and Ly6G (1:500) at 4°C overnight, the sections were washed 3 times with PBS and incubated with the secondary antibodies (1:1,500) for 2 hours at room temperature (RT). After another 3 washes with PBS, the coverslips were mounted using mounting medium containing 4′,6-diamidin-2-phenylindol (DAPI) and visualized on an Olympus confocal laser scanning microscope (FluoView FV1000) using a 20x air objective with a tile and stitching mode. Images were identically acquired and processed with Fiji/ImageJ to calculate the percentage of H4Cit and Ly6G positive area.

### Mouse Neutrophil Isolation

Blood was collected from the retro-orbital plexus of anesthetized mice in 1 mL of ethylenediaminetetraacetic acid (EDTA) anticoagulated buffer supplemented with 1% endotoxin-free BSA in sterile PBS, and peripheral blood neutrophils were subsequently isolated. Bone marrow–derived neutrophils were obtained by flushing the mouse femur 3-4 times with phenol red-free RPMI 1640 medium supplemented with 10 mM HEPES. The bone marrow–cell suspension was strained using a 40 µm cell strainer, and cells were pelleted by 10 minutes of 500 x g centrifugation before finally being resuspended in PBS.

Subsequently, peripheral or bone marrow–derived neutrophils were isolated by Percoll gradient centrifugation, as described elsewhere (7). Neutrophils were then resuspended in phenol red-free RPMI 1640 medium supplemented with 10 mM HEPES, and cell purity was assessed by Wright-Giemsa stain. After the neutrophil count was determined, the required cell density was adjusted by adding HEPES supplemented RPMI 1640 medium.

### Human Neutrophil Isolation

The experimental procedure was approved by the Office of Clinical Investigations at Boston Children’s Hospital (protocol number IRB-P00003283). Informed consent was provided by donors. Blood was drawn from healthy donors in EDTA-coated vacutainers, and blood samples were de-identified prior to isolation. Neutrophils were isolated using gradient centrifugation, as described elsewhere (7). Cells were resuspended in phenol red free RPMI 1640 medium supplemented with HEPES, assessed for purity by Wright-Giemsa stain, and adjusted to the desired cell density.

### Cell Culture of iBMDM Cells

Immortalized mouse bone marrow**–**derived macrophages (iBMDM) (42) were cultured in Dulbecco’s modified Eagle’s medium (DMEM) containing 10% fetal bovine serum (FBS), 1% penicillin/streptomycin and supplemented with L-glutamine and sodium pyruvate. Cells were split every 3 days in a 1:10 ratio by detaching them in PBS (pH 7.4) containing 2 mM EDTA.

### Gene Editing of iBMDM Cells Overexpressing PAD4

PAD4 overexpressing construct was generated using a murine PAD4-mScarlet vector, which was produced by inserting mScarlet cDNA into the pLV-eGFP plasmid (a gift from Pantelis Tsoulfas, Addgene no: 36083) between XbaI and BamHI sites. The mouse full-length PAD4 insert was amplified and ligated between the AgeI and SalI sites rom he cDNA using the following primers: forward primer of 5’-ACCTCCATAGAAGACACCGACTCTAGAATGGCCCAAGGCGCGGTGATCCA-3’, and reverse primer of 5’-CTTGCTCACCATTGAGCCGCTACCGGTGGGCACCATGTGCCACCACTTGA-3’.

### Generation of Stable Cell Lines

Stable PAD4-mScarlet overexpressing iBMDM cell lines were generated by a lentiviral transfection approach. To this end, HEK293T cells were co-transfected with 1 μg of pLV plasmid containing the corresponding gene, 750 ng psPAX2 packaging plasmid, and 250 ng pMD2.G envelope plasmid (both plasmids were a gift from Didier Trono, Addgene no: 12260 and 12259, respectively) on day 0 and incubated overnight. On day 1, the medium was removed, replenished with 1 mL fresh medium, and the cells were incubated for another day. On day 2, the supernatant containing the virus was filtered using a 0.45 μm filter and used directly to infect iBMDM cells by spinfection at 2,500 x g for 90 minutes at RT using 8 μg/mL polybrene. Subsequently, cells were incubated in the corresponding culture medium for 24 hours, and positively infected cells were sorted by flow cytometry using mScarlet (PAD4) wavelengths. Positive cell colonies were validated at protein and functional level.

### Western Blot

For western blot analysis, cells were lysed in RIPA buffer supplemented with protease and phosphatase inhibitors, according to the manufacturer’s protocol, and incubated for 30 minutes on ice. After centrifugation at 20,000 x g for 15 minutes at 4°C, the protein concentration in the supernatant was measured using Bradford reagent. The protein samples were then denatured in LDS buffer and reducing agent for 5 minutes at 95°C. Lysates were separated by 4-12% Bis-Tris gradient gels, and proteins were electrotransferred on a PVDF membrane using the iBlot system. Membranes were blocked with 5% BSA in TBS-T buffer (0.05% Tween-20 in 1x TBS) for 1 hour at RT and incubated overnight at 4°C with anti ASC (1:800), anti NLRP3 (1:1,000), anti caspase-1 (1:1,000), anti PAD4 (1:200) or anti GAPDH (1:5,000) antibodies. For probing with the PAD4 antibody, the membranes were stripped for 20 minutes at RT using 0.5 M NaOH solution, blocked with 5% BSA TBS-T buffer, and incubated for 4 hours at RT using a custom-made mouse-specific PAD4 antibody (Thermo Fisher Scientific) directed against mouse PAD4 peptide DKEDPQASGMDFEDDKILD that does not cross react with mouse PAD2. After incubation with primary antibodies, membranes were washed 3 times with TBS-T buffer before incubation with HRP-conjugated secondary antibodies (1:10,000) and were incubated for 2 hours at RT. Then the membranes were washed 3 more times with TBS-T and subsequently probed with enhanced chemiluminescence (ECL) detection solution.

### IL-1ß ELISA

IL-1ß was measured according to the manufacturer’s instruction. A 96-well plate was coated with the capturing antibody overnight at 4°C. The following day, the plate was washed 3 times with buffer (PBS + 0.05% Tween-20) and then blocked at RT for 1 hour under gentle shaking. Subsequently, after 3 further washing steps, 100 µL of iBMDM supernatant or standard solution was added per well and incubated for 2 hours at RT under gentle shaking. The plate was again washed 3 times before the addition of the detection antibody. After incubation of the detection antibody for 1 hour at RT, avidin-HRP was added and incubated for 0.5 hours at RT. After final washing steps, TMB solution was supplemented, and 2N H_2_SO_4_ was used as stop solution. The absorbance was read at 450 and 570 nm. To obtain final values, the 570 nm values were subtracted from the 450 nm values.

### qRT-PCR

Total RNA was extracted using the PureLink™ RNA Mini Kit (ThermoFisher) according to the manufacturer’s instructions. Complementary DNA (cDNA) was synthesized using All-In-One RT MasterMix following the manufacturer’s instructions. Quantitative PCR of specific genes was performed using SYBR Green SuperMix in the StepOnePlus RealTime PCR System. Cycling conditions were as follows: initial denaturation at 95°C for 2 minutes, followed by 40 cycles of 95°C for 15 sec, 55°C for 30 sec, and 70°C for 15 sec. For amplification, the following primer pairs were used (5`- >3`orientation): β-actin: fwd: CGGTTCCGATGCCCTGAGGCTCTT; rev: CGTCACACTTCATGATGGAATTGA for isolated peripheral neutrophils and fwd: CATTGCTGACAGGATGCAGAAGG; rev: TGCTGGAAGGTGGACAGTGAGG for iBMDM cells; ASC: fwd: CAGAGTACAGCCAGAACAGGACAC, rev: GTGGTCTCTGCACGAACTGCCTG; NLRP3: fwd: GTTCTGAGCTCCAACCATTCT, rev: CACTGTGGGTCCTTCATCTTT; IL1ß: fwd: TGGACCTTCCAGGATGAGGACA; rev: GTTCATCTCGGAGCCTGTAGTG​.

To confirm the equal RNA input, β-actin mRNA expression and the relative expression of inflammasome mRNA were calculated with the △△Ct method. Specificity of the amplification was checked by melting curve analysis, and data were recorded and analyzed using StepOne Software v2.1.

### *In Vitro* NET Assay

1.5x10^4^ mouse or human neutrophils per well were resuspended in HEPES supplemented phenol red-free RPMI 1640 medium and plated in a 96-well plate. After allowing the cells to adhere for 30 minutes at 37°C and 5% CO_2_ in the absence or presence of 1 µM MCC950, the cells were stimulated with vehicle control, nigericin (15 µM), or ionomycin (4 µM) for 4 hours. Fixation was performed in 2% PFA containing Hoechst 33342 (1:10,000) at 4°C overnight. Cells were washed 3 times with PBS the next day before imaging on a Zeiss Axiovert 200M microscope. The percentage of NETs was analyzed from 8 non-overlapping and randomized visual fields per well by quantifying cells with a web-like chromatin structure and positive citrullinated histone H4 staining. The average percentage of NETing cells was taken from duplicates in each experiment.

### Immunofluorescence Staining of ASC Speck in Neutrophils

6x10^4^ mouse or 1x10^5^ human neutrophils per condition were plated on a sterilized coverslip in a 6-well plate and allowed to adhere for 30 min at 37°C and 5% CO_2_ before stimulating the cells with nigericin (15 µM), ionomycin (4 µM), or PMA (50 nM) for 4 hours. The cells were fixed with 4% PFA for 1 hour at RT, washed once with PBS, permeabilized (0.1% Triton X-100, 0.1% sodium citrate) for 10 minutes at 4°C, and incubated with blocking buffer (2.5% BSA, 0.5% Tween-20 in 1x PBS) at 37°C for 1 hour. Samples were incubated at 4°C ON with the primary antibodies against ASC (1:800, mouse neutrophils or 1:200, human neutrophils) and subsequently washed 3 times with PBS before incubation with the secondary antibody (1:1,500) for 2 hours at RT. After another 3 washing steps with PBS, the coverslips were mounted using mounting medium containing DAPI and visualized on a confocal Nikon Eclipse Ti2 microscope using a 60x oil immersion objective (mouse neutrophils) or an Olympus confocal laser scanning microscope FluoView (FV1000) using a 40x air objective (human neutrophils). Images were identically acquired and processed with Fiji/ImageJ. ASC speck frequency was determined by capturing 37 Z-stacks of 0.1625 µm size from 6 by 6 tiles on a Nikon Eclipse Ti2 A1R confocal microscope (mouse neutrophils) and by 5 non-overlapping and randomized visuals fields on an Olympus FluoView (FV1000) confocal microscope (human neutrophils) in the center of the coverslip. The percentage of neutrophils forming ASC speck was quantified.

### Immunofluorescence Staining of ASC Speck in iBMDM

iBMDMs were plated on a 35 mm glass-bottom dish and primed for 4 hours with LPS from E.coli (1 µg/mL) at 37°C and 5% CO_2_ before stimulating the cells with nigericin (20 µM) for 30 minutes. iBMDMs were fixed in 3% PFA containing 1:10,000 Hoechst 33342 for 30 minutes at RT, washed twice with PBS for 5 minutes, and permeabilized (0.1% Triton X-100, 0.1% sodium citrate) for 7 minutes at RT, followed by a washing step. Afterwards, cells were blocked for 1 hour at RT with blocking buffer (3% BSA in PBS) and subsequently incubated with the primary antibody ASC (1:1,000) overnight. Followed by extensive washing steps, cells were incubated with the corresponding secondary antibody (1:2,000) the next day for 2 hours at RT. Finally, cells were visualized with an Olympus confocal laser scanning microscope FluoView (FV1000). Images were captured using a 60x water immersion objective with Olympus FluoView version 3.0 viewer software. The images were identically acquired and processed using ImageJ software, and the percentage of iBMDMs developing an ASC speck was quantified.

### Time-Lapse Visualization by Spinning Disc Confocal and DIC Microscopy

Time-lapse microscopy was performed using isolated peripheral neutrophils from *Nlrp3^+/+^* and *Nlrp3^–/–^* mice. To this end, 1x10^6^ mouse neutrophils were stained for 30 min at 37°C and 5% CO_2_ using 2 µM SiR-DNA to visualize chromatin and 1 µM ER-tracker red dye to visualize the endoplasmic reticulum and nuclear envelope. Subsequently, cells were washed and resuspended in 300 µL of imaging media (phenol red-free RPMI 1640, 25 mM HEPES, 1% penicillin/streptomycin) before the cell suspension was added and allowed to adhere for 5 min in a non-coated, 24-well glass-bottom plate located on a 37°C pre-warmed microscope stage. 3-10 random fields per well were visualized using a Nikon Eclipse Ti2 microscope equipped with Perfect Focus™, a Yokogawa CSU-X1 spinning disc scanhead, a Nikon motorized stage with XY linear encoders containing a Nano-Z100 piezo insert, and a Hamamatsu Orca-flash 4.0 v3 camera with a Plan Apo Tirf 60x oil 1.49 NA DIC Nikon objective lens. Confocal and DIC images were acquired every 2 minutes for the first 80 minutes and every 5 minutes for the rest of the visualization up to 4 hours. Three images were acquired of unstimulated cells, followed by addition of imaging medium containing ionomycin to achieve a final ionomycin concentration of 4 µM.

### Statistical Analysis

All data are presented as mean ± standard error of the mean (SEM). Statistical analysis was performed using GraphPad Prism. Significance was tested with unpaired t-test or, for experiments with more than two groups, with two-way ANOVA multiple comparison test. p<0.05 was considered statistically significant.

## Results

### Neutrophil Activation Induces NLRP3-Dependent ASC Speck Formation Under Sterile Conditions

**Figure 1:**
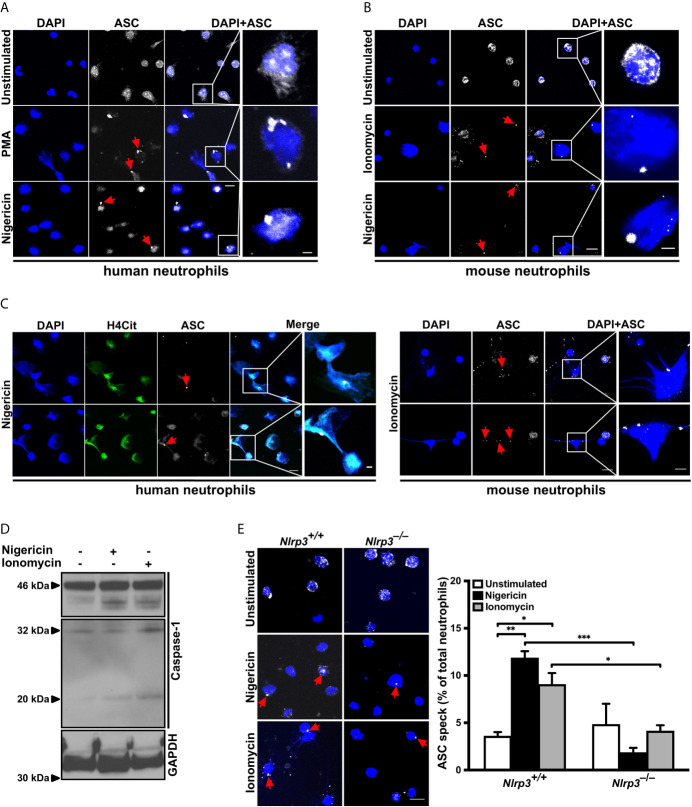
NLRP3 inflammasome-dependent ASC speck formation and release on NETs by stimulated neutrophils **(A)** Confocal microscopy images of immunostained human neutrophils in the absence (unstimulated) or presence of PMA (50 nM) or nigericin (15 µM) for 4 hours. Blue, DNA (DAPI); grey, ASC antibody staining. Red arrows indicate ASC speck. Scale bar equals 10 µm in the overview and 2.5 µm in the zoom panel. Representative of n=4 experiments. **(B)** Confocal microscopy images of immunostained mouse neutrophils in the absence (unstimulated) or presence of ionomycin (4 µM) or nigericin (15 µM) for 4 hours. Blue, DNA (DAPI); grey, ASC antibody staining. Red arrows indicate ASC speck. Scale bar equals 10 µm in the overview and 2.5 µm in the zoom panel. Representative of n=5 experiments. **(C)** Confocal microscopy images of ASC speck formation in NETting cells and associated with NETs in human (left panel) or mouse neutrophils (right panel) in the presence of nigericin (15 µM) or ionomycin (4 µM). Blue, DNA (DAPI); green, H4Cit antibody stain; grey, ASC antibody staining. Red arrows indicate ASC speck. Scale bar equals 10 µm in the overview and 2.5 µm in the zoom panel. Representative of n=4-5 experiments. **(D)** Western blot of caspase-1 cleavage (antibody clone: Casper-1) in neutrophils from wild-type mice in the absence or presence of nigericin (15 µM) or ionomycin (4 µM) for 0.5 hours. Representative of n=3 experiments. **(E)** Representative confocal microscopy images (left panel) and arithmetic means ± SEM (right panel; n=4-5 mice) of ASC speck formation in neutrophils from wild-type (*Nlrp3^+/+^*) or *Nlrp3^–/–^* mice in the absence (unstimulated, open bars) or presence of nigericin (15 µM, black bars) or ionomycin (4 µM, grey bars) for 4 hours. Blue, DNA (DAPI); grey, ASC antibody staining. Red arrows indicate ASC speck. Scale bar equals 10 µm. *p<0.05, **p<0.01 and ***p<0.001.

NLRP3 is expressed in human and mouse neutrophils (22, 23). To investigate whether NLRP3 inflammasome assembles in neutrophils under sterile conditions, human peripheral neutrophils were activated with PMA or nigericin for 4 hours. Both agents induced inflammasome assembly, observed in a subset of cells by immunostaining for ASC speck formation, which was absent in unstimulated neutrophils (**Figure 1A**). Like human neutrophils, isolated peripheral mouse neutrophils displayed ASC speck formation when stimulated with ionomycin or nigericin (**Figure 1B**) and stained with an antibody specific for ASC (**Figure S1**) subsequently. Interestingly, ASC speck formation occurred in NET-forming human or mouse neutrophils upon stimulation with nigericin or ionomycin, respectively (**Figure 1C**). After ionomycin- or nigericin- stimulated NET release, a fraction of neutrophils undergoing NETosis also showed ASC speck or its fragments associated with the expelled extracellular chromatin (**Figure 1C**; red arrowheads).

Next, since activation of pro-caspase-1 upon ASC speck formation is an established hallmark of inflammasome activation, caspase-1 cleavage products were investigated in mouse neutrophils. Stimulation with nigericin or ionomycin caused generation of the characteristic p32 and p20 caspase-1 fragments (**Figure 1D**).

To confirm that the observed ASC speck formation in neutrophils is due to NLRP3 assembly, we treated circulating neutrophils from wild-type (*Nlrp3^+/+^*) and NLRP3-deficient (*Nlrp3^–/–^*) mice with nigericin or ionomycin. As shown in **Figure 1E**, nigericin or ionomycin stimulation induced ASC speck formation in approximately 10% of wild-type neutrophils. By contrast, this activation-dependent increase in ASC speck formation was significantly reduced in neutrophils from *Nlrp3^–/–^* mice, indicating that the majority of observed ASC speck in the mouse neutrophils was part of the NLRP3 inflammasome (**Figure 1E**).

Taken together, these results demonstrate that neutrophils can assemble a physiologically active inflammasome/ASC speck in the absence of bacteria or LPS.

### PAD4 Supports ASC Speck Formation by Regulation of ASC and NLRP3 Protein Levels

ASC speck forms in stimulated neutrophils, where PAD4 is a prerequisite for NET formation (15). We wondered whether PAD4 may promote ASC speck assembly as well. Since PAD4 can be synthesized by many cell types and can be found in plasma, a hematopoietic cell-specific *Padi4* knockout mouse was generated using Cre-Lox recombination by intercrossing *Padi4^fl/fl^* with *Vav1-iCre* mice (**Figure S2A**). The resulting PAD4 wild-type (*Padi4^fl/fl^*) and hematopoietic cell-specific knockout (*Padi4^Vav1Cre/+^*) mice showed no differences in blood cell counts (**Figure S2B**) and, in agreement with global *Padi4^–/–^* mice, *Padi4^Vav1Cre/+^* mice displayed significantly reduced NETosis (**Figure S2C**).

**Figure 2:**
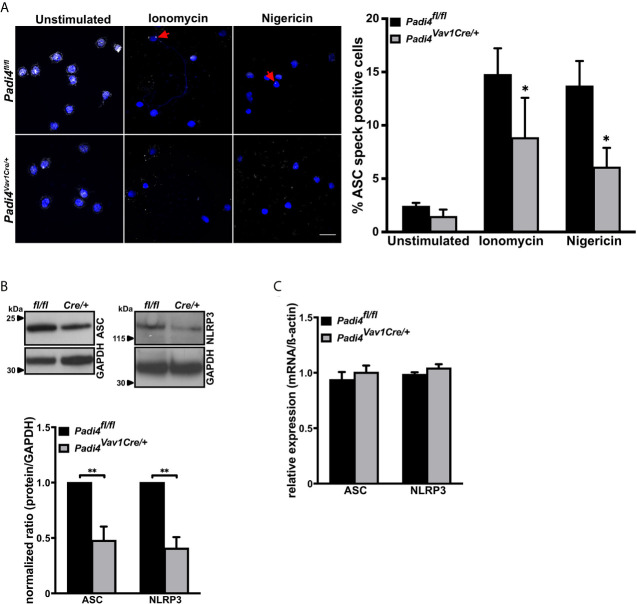
ASC speck formation in neutrophils is, in part, directed by PAD4-dependent regulation of ASC and NLRP3 protein levels **(A)** Representative confocal microscopy images (left panel) and arithmetic means ± SEM (right panel, n=4-6 mice) of ASC speck formation in neutrophils from wild-type (*Padi4^fl/fl^*, black bars) or hematopoietic cell-specific Padi4-deficient mice (*Padi4^Vav1Cre/+^*, grey bars) in the absence (unstimulated) or presence of ionomycin (4 µM) or nigericin (15 µM) for 4 hours. Blue, DNA (DAPI); grey, ASC antibody staining. Red arrows indicate ASC speck. Scale bar equals 10 µm. *p<0.05. **(B)** Representative western blots (upper panel) and arithmetic means ± SEM (lower panel, n=4 mice) of ASC and NLRP3 protein levels in naive neutrophils from wild-type (*Padi4^fl/fl^*, black bars) or hematopoietic specific Padi4-deficient mice (*Padi4^Vav1Cre/+^*, grey bars). **p<0.01. **(C)** Arithmetic means ± SEM (n=3 mice) of relative mRNA levels in naive neutrophils from wild-type (*Padi4^fl/fl^*, black bars) or hematopoietic specific Padi4-deficient mice (*Padi4^Vav1Cre/+^*, grey bars).

Immunostaining of unstimulated *Padi4^fl/fl^* or *Padi4^Vav1Cre/+^* neutrophils detected ASC speck formation in approximately 2% of the cells. However, nigericin or ionomycin stimulation resulted in ASC speck formation in about 15% of *Padi4^fl/fl^* cells, while only 6-8% of neutrophils from *Padi4^Vav1Cre/+^* mice showed ASC speck formation (**Figure 2A**), which shows that neutrophil PAD4 is needed to fully stimulate ASC speck assembly. Since no LPS pretreatment was needed for induction of ASC speck formation in neutrophils, we decided to compare the protein levels of NLRP3 and ASC in these cells. Interestingly, peripheral neutrophils lacking PAD4 (*Padi4^Vav1Cre/+^*) displayed decreased ASC and NLRP3 protein levels when compared with PAD4-positive neutrophils from *Padi4^fl/fl^* mice (**Figure 2B**), while LPS pretreatment equalized the ASC and NLRP3 protein levels in these neutrophils (**Figure S2D**). Although protein levels of the NLRP3 inflammasome are transcriptionally regulated by NFκB in macrophages (43), neutrophils from *Padi4^fl/fl^* and *Padi4^Vav1Cre/+^* mice showed no difference in NLRP3 and ASC mRNA levels (**Figure 2C**).

These results indicate that PAD4 has the ability to upregulate NLRP3 inflammasome components in a post-transcriptional manner without *de novo* mRNA synthesis in neutrophils.

### Overexpresion of PAD4 Bypasses LPS Priming During NLRP3 Inflammasome Assembly in Bone Marrow–Derived Macrophages

To study whether PAD4 also plays a role in ASC speck formation in other cell types, we prepared primary bone marrow–derived macrophages (BMDM) from wild-type and *Padi4^Vav1Cre/+^* mice. While inhibition of citrullinating activity of several PAD enzymes by Cl-amidine almost completely abrogated ASC speck formation in primary wild-type BMDMs (**Figures S3A, B**), there was no statistically significant difference in ASC speck formation in primary bone marrow–derived macrophages from *Padi4^Vav1Cre/+^* mice when compared with *Padi4^fl/fl^* BMDMs after LPS and nigericin exposure (**Figure S3C**). This observation confirms a previous report (44) and indicates that in mouse macrophages, as in neutrophils, citrullination is necessary for ASC speck formation. However, other PAD enzymes may compensate for the lack of PAD4 in macrophages.

**Figure 3:**
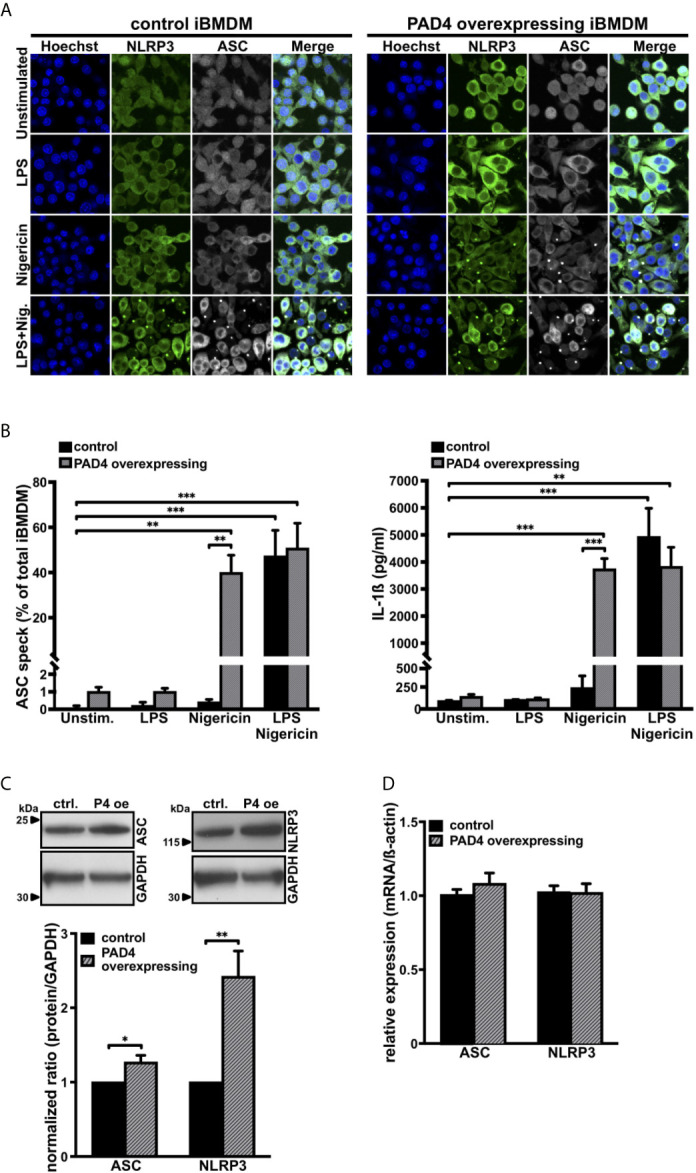
PAD4 overexpression leads to priming-independent ASC speck formation and IL-1ß secretion with an increase in ASC and NLRP3 protein levels in immortalized bone marrow–derived murine macrophages **(A)** Confocal microscopy images of immunostained native mouse immortalized bone marrow–derived macrophages (iBMDM; control, left panel) and PAD4 overexpressing iBMDM (right panel) in the absence (unstimulated) or presence of LPS only (1 µg/mL), nigericin only (15 µM), or LPS and nigericin (15 µM, 0.5 hours). Blue, DNA (Hoechst); green, NLRP3 antibody staining; grey, ASC antibody staining. Scale bar equals 5 µm. Representative of n=4 experiments. **(B)** Arithmetic means ± SEM (n=4) of percentage of ASC speck formation (left panel) and IL-1ß production (right panel) in native iBMDM (control, black bars) or PAD4 overexpressing iBMDM (grey shaded bars) in the absence (unstim.) or presence of LPS only (1 µg/mL), nigericin only (15 µM), or LPS (1 µg/mL) and nigericin (15 µM, 0.5 hours). **p<0.01 and ***p<0.001. **(C)** Representative western blots (upper panel) and arithmetic means ± SEM (lower panel, n=4) of ASC and NLRP3 protein levels in native iBMDM (ctrl.) or PAD4 overexpressing (P4 oe) iBMDM. *p<0.05 and **p<0.01. **(D)** Arithmetic means ± SEM (n=4) of relative mRNA levels in native iBMDM (control) or PAD4 overexpressing iBMDM.

To further investigate the role of PAD4 in NLRP3 inflammasome assembly in macrophages, we generated PAD4 overexpressing immortalized bone marrow–derived macrophages (iBMDM). While these cells exhibited a 4-fold increased PAD4 protein level compared with wild-type iBMDM (**Figures S4A, B**), PAD4 overexpression had no effect on IL-1ß mRNA levels in iBMDM (**Figure S4C**). Although *in vitro* stimulation of NLRP3 assembly in iBMDMs requires pretreatment with LPS and subsequent stimulation with nigericin, we observed that nigericin alone was able to induce ample ASC speck formation and IL-1ß production in PAD4 overexpressing iBMDMs (**Figures 3A, B**). Stimulation of control iBMDMs (empty vector-treated cells) with nigericin or LPS alone did not induce ASC speck formation or IL-1ß production (**Figures 3A, B**). In agreement with the observation in neutrophils, PAD4 overexpression in iBMDMs resulted in significantly increased NLRP3 protein levels and a small but significant increase in ASC levels when compared with empty vector expressing cells, without a significant change in the corresponding mRNA levels (**Figures 3C, D**).

These results show the importance of PAD4 activity in inflammasome/ASC speck assembly in iBMDM cells under sterile conditions.

### NLRP3 Inflammasome Supports NETosis by Promoting Nuclear Envelope and Plasma Membrane Breakdown

**Figure 4:**
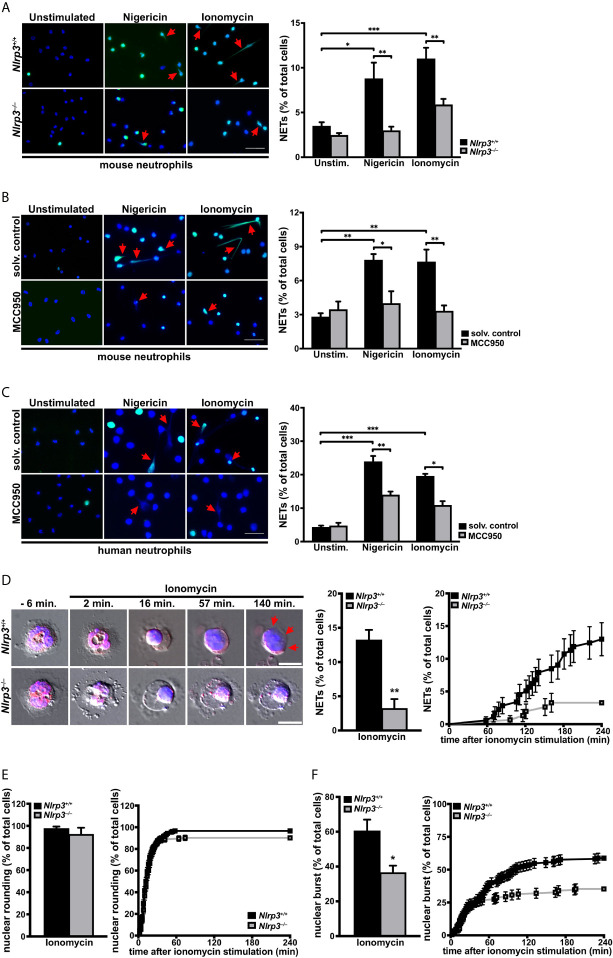
The NLRP3 inflammasome promotes activation-dependent NET formation through nuclear envelope and plasma membrane breakdown in primary neutrophils. **(A)** Overview (left panel) and arithmetic means ± SEM (right, n=5-7 mice) of NET formation by neutrophils from wild-type (*Nlrp3^+/+^*, black bars) or *Nlrp3^–/–^* mice (grey bars) in the absence (unstimulated) or presence of nigericin (15 µM) or ionomycin (4 µM) for 4 hours. Blue, DNA (DAPI); green, H4Cit antibody stain. Red arrows indicate NETs. Scale bar equals 50 µm. *p<0.05, **p<0.01 and ***p<0.001. **(B)** Overview (left panel) and arithmetic means ± SEM (right, n=5-6 mice) of NET formation in untreated (solvent control, black bars) or MCC950 pretreated (1 µM, grey bars) mouse neutrophils in the absence (unstimulated) or presence of nigericin (15 µM) or ionomycin (4 µM) for 4 hours. Blue, DNA (DAPI); green, H4Cit antibody stain. Red arrows indicate NETs. Scale bar equals 50 µm. *p<0.05 and **p<0.01. **(C)** Overview (left panel) and arithmetic means ± SEM (right, n=4-8 donors) of NET formation in untreated (solvent control, black bars) or MCC950 pretreated (1µM, grey bars) human neutrophils in the absence (unstimulated) or presence of nigericin (15 µM) or ionomycin (4 µM) for 4 hours. Blue, DNA (DAPI); green, H4Cit antibody stain. Red arrows indicate NETs. Scale bar equals 50 µm. *p<0.05, **p<0.01 and ***p<0.001. **(D)** Representative time-lapse differential interference contrast (DIC) spinning-disk confocal microscopy images at indicated time intervals (left panel) and arithmetic means ± SEM (center/right panel; n=5 mice) of percentage of total (middle) and time course (right) of plasma membrane rupture (NETosis) in neutrophils from wild-type (*Nlrp3^+/+^*, black bars) or *Nlrp3^–/–^* mice (grey bars) in the presence of ionomycin (4 µM). Blue, DNA (siR-DNA); red, nuclear envelope (ER-tracker). Red arrows indicate area of plasma membrane rupture. Scale bar equals 5 µm. **p<0.01. **(E)** Arithmetic means ± SEM (n=5 mice) of percentage of total (left panel) and time course (right panel) of nuclear rounding in neutrophils from wild-type (*Nlrp3^+/+^*, black bars) or *Nlrp3^–/–^* mice (grey bars) in the presence of ionomycin (4 µM). **(F)** Arithmetic means ± SEM (n=5 mice) of percentage of total (left panel) and time course (right panel) of nuclear envelope rupture in neutrophils from wild-type (*Nlrp3^+/+^*, black bars) or *Nlrp3^–/–^* mice (grey bars) in the presence of ionomycin (4 µM). *p<0.05.

Since ASC speck formation was observed in neutrophils forming NETs, we addressed the importance of the NLRP3 inflammasome assembly on NETosis. To this end, we examined the effects of inflammasome perturbations on NETosis in *in vitro* assays. Neutrophils isolated from *Nlrp3^–/–^* mice showed significantly decreased NETosis after nigericin or ionomycin stimulation when compared with neutrophils from wild-type mice (**Figure 4A**). The role of NLRP3 was further substantiated both in mouse and human neutrophils by pharmacological inhibition of the NLRP3 inflammasome. Pretreatment for 30 minutes with the specific small molecule NLRP3 inhibitor MCC950 (33) resulted in significantly decreased NETosis after nigericin or ionomycin stimulation than observed in vehicle-treated neutrophils (**Figures 4B, C**). Thereby, the inhibition was more pronounced in mouse than human neutrophils, but was statistically significant in both cases. In line with results from NLRP3 perturbations, we found that pharmacological inhibition of the NLRP3 inflammasome effector molecule caspase-1 also revealed markedly reduced NETosis in human neutrophils (**Figure S5**), indicating that NLRP3-mediated caspase-1 activation is needed for efficient NETosis.

To determine the underlying NLRP3-dependent cellular processes in NETosis, we performed time-lapse microscopy of ionomycin-stimulated neutrophils from *Nlrp3^+/+^* and *Nlrp3^–/–^* mice. Spinning disk confocal and DIC microscopy of cells stained with SiR-DNA as a marker of chromatin and ER-tracker as a marker of the endoplasmic reticulum (ER) and nuclear envelope confirmed a significantly impaired NET formation in neutrophils from *Nlrp3^–/–^* mice when compared with wild-type neutrophils. While neutrophils from *Nlrp3^+/+^* mice showed a robust breakage of the plasma membrane (NETosis) starting 60 minutes after stimulation, *Nlrp3-*deficient neutrophils displayed four-fold reduced plasma membrane rupture (**Figure 4D**). While nuclear rounding, another described cellular characteristic of NETosis (45), occurred both in neutrophils from *Nlrp3^+/+^* and *Nlrp3^–/–^* mice, most *Nlrp3^–/–^* neutrophils arrested the NETosis process at this stage (**Figure 4E** and **Videos S1, S2**). Accordingly, the rupture of the nuclear envelope was significantly impaired in neutrophils from *Nlrp3^-/-^* mice when compared with wild-type controls (**Figure 4F**).

The above observations demonstrate an important role of the NLRP3 inflammasome in nuclear envelope and plasma membrane breakdown after sterile inflammation, thus pointing to a central role of the NLRP3 inflammasome in NETosis.

### NLRP3 Inflammasome Promotes NETosis *In Vivo* and Supports Venous Thrombus Progression in Mice

**Figure 5:**
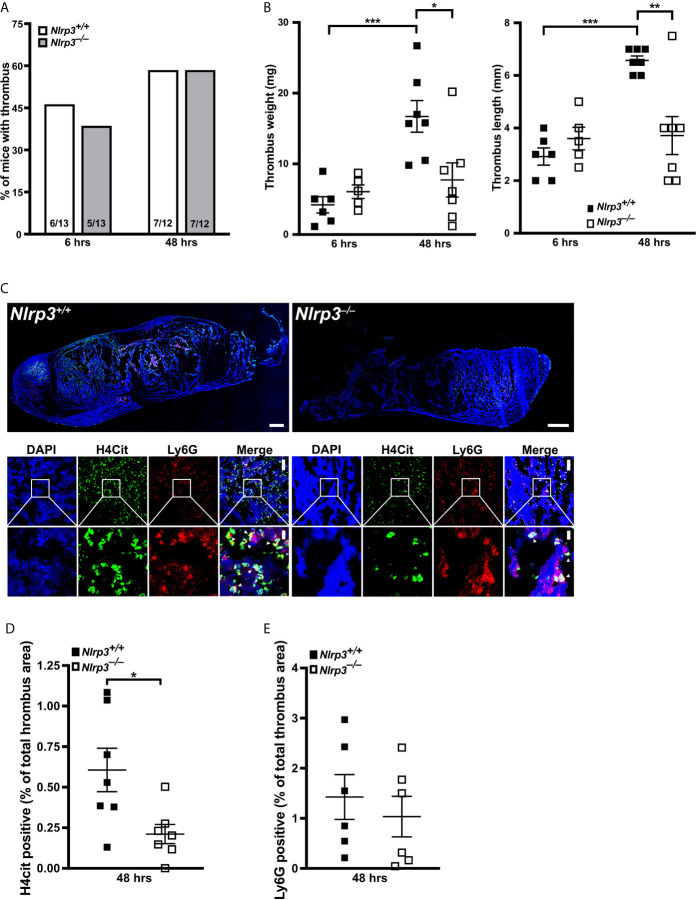
NLRP3 deficiency decreases NETosis and thrombus growth in a stenosis-induced model of deep vein thrombosis. **(A)** Thrombus incidence in wild-type (*Nlrp3^+/+^*, open bars) or *Nlrp3^–/–^* mice (grey bars) after 6 or 48 hours of stenosis of the inferior vena cava (IVC). **(B)** Thrombus weight (left panel) and thrombus length (right panel) of thrombi from wild-type (*Nlrp3^+/+^*, black) or *Nlrp3^–/–^* mice (white) after 6 or 48 hours of stenosis of the IVC. Each dot represents a thrombus. *p<0.05, **p<0.01 and ***p<0.001. **(C)** Representative composite images of thrombi by confocal microscopy (upper panels) and zoom images (lower panels) of thrombi from wild-type (*Nlrp3^+/+^*) or *Nlrp3^–/–^* mice after 48 hours of stenosis of the IVC. Blue, DNA (DAPI); green, H4Cit antibody stain; red, Ly6G antibody staining. Overview images were composed of several photographs. Scale bar equals 300 µm (thrombus overview), 50 µm (upper panel) or 10 µm (lower panel) in the zoom panels. **(D)** Percentage of thrombus area covered by H4Cit in thrombi from wild-type (*Nlrp3^+/+^*, black) or *Nlrp3^–/–^* mice (white) after 48 hours of stenosis of the IVC. Each dot represents a thrombus. *p<0.05. **(E)** Percentage of thrombus area covered by Ly6G positive cells in thrombi from wild-type (*Nlrp3^+/+^*, black) or *Nlrp3^–/–^* mice (white) after 48 hours of stenosis of the IVC. Each dot represents a thrombus.

We next sought to verify that NLRP3 also regulates NETosis in deep vein thrombosis (DVT) to unravel the physiological importance of our findings. To this end, we applied a mouse model of stenosis-induced DVT, which is an acknowledged murine model for sterile thrombo-inflammation, in wild-type (*Nlrp3^+/+^*) and *Nlrp3^–/–^* mice. In our previous work with PAD4-deficient mice, we noted that NETs likely stabilize the thrombus, since lack of NETosis displayed a more important phenotype at later timepoints of venous thrombus progression (14). Therefore, we induced vascular stenosis for 6 or 48 hours to monitor thrombus progression. While the incidence of formed thrombi in *Nlrp3^+/+^* and *Nlrp3^–/–^* mice was similar at both time points (**Figure 5A**), there was a significant NLRP3-dependent difference in thrombus progression. Interestingly, thrombus length and weight did not differ between *Nlrp3^+/+^* and *Nlrp3^–/–^* mice 6 hours after stenosis. By contrast, a significant reduction in thrombus size was observed in *Nlrp3^–/–^* mice after 48 hours when compared with *Nlrp3^+/+^* mice. Furthermore, although the thrombi from *Nlrp3^+/+^* mice increased in weight and length over the 48-hour period, thrombi from NLRP3-deficient mice reached their final small size already after only 6 hours, resulting in smaller thrombi in NLRP3^–/–^ mice when compared with thrombi from wild-type mice (**Figure 5B**). This observation points to a role of NLRP3 in venous thrombosis progression under sterile conditions.

Since thrombus growth in the DVT model is substantially dependent on NETosis, we finally investigated the density of citrullinated histone H4 (H4Cit) in thrombi from *Nlrp3^+/+^* and *Nlrp3^–/–^* mice after 48 hours of stenosis as a direct marker for the presence of NETs. As shown in **Figures 5C, D**, NET density, and thus their formation, was significantly reduced in thrombi from *Nlrp3^–/–^* mice when compared with wild-type controls (**Figures 5C, D**). Neutrophil density in the corresponding thrombi were comparable between *Nlrp3^+/+^* and *Nlrp3^–/–^* mice (**Figure 5E**), emphasizing an NLRP3-dependent enhancement of NETosis with subsequent increase in venous thrombus growth *in vivo*.

Our results demonstrate that, at least in the mouse, NLRP3 inflammasome is critical for NETosis *in vivo*.

## Discussion

NETs and NLRP3 inflammasome are formed in a similar set of human disorders and infectious diseases. Among these pathologies, noninfectious diseases such as cancer, immunothrombosis, myocardial infarction, and stroke are the main causes of death (46). In particular, it is known that neutrophils and NETosis are major inducers of venous thrombosis (47) and hypoxia-induced venous thrombosis is linked to elevated IL-1ß and NLRP3 levels in thrombi (37, 48). However, it was not known if canonical inflammasome assembly takes place in neutrophils under these noninfectious conditions, and if inflammasome assembly contributes to NETosis.

**Figure 6:**
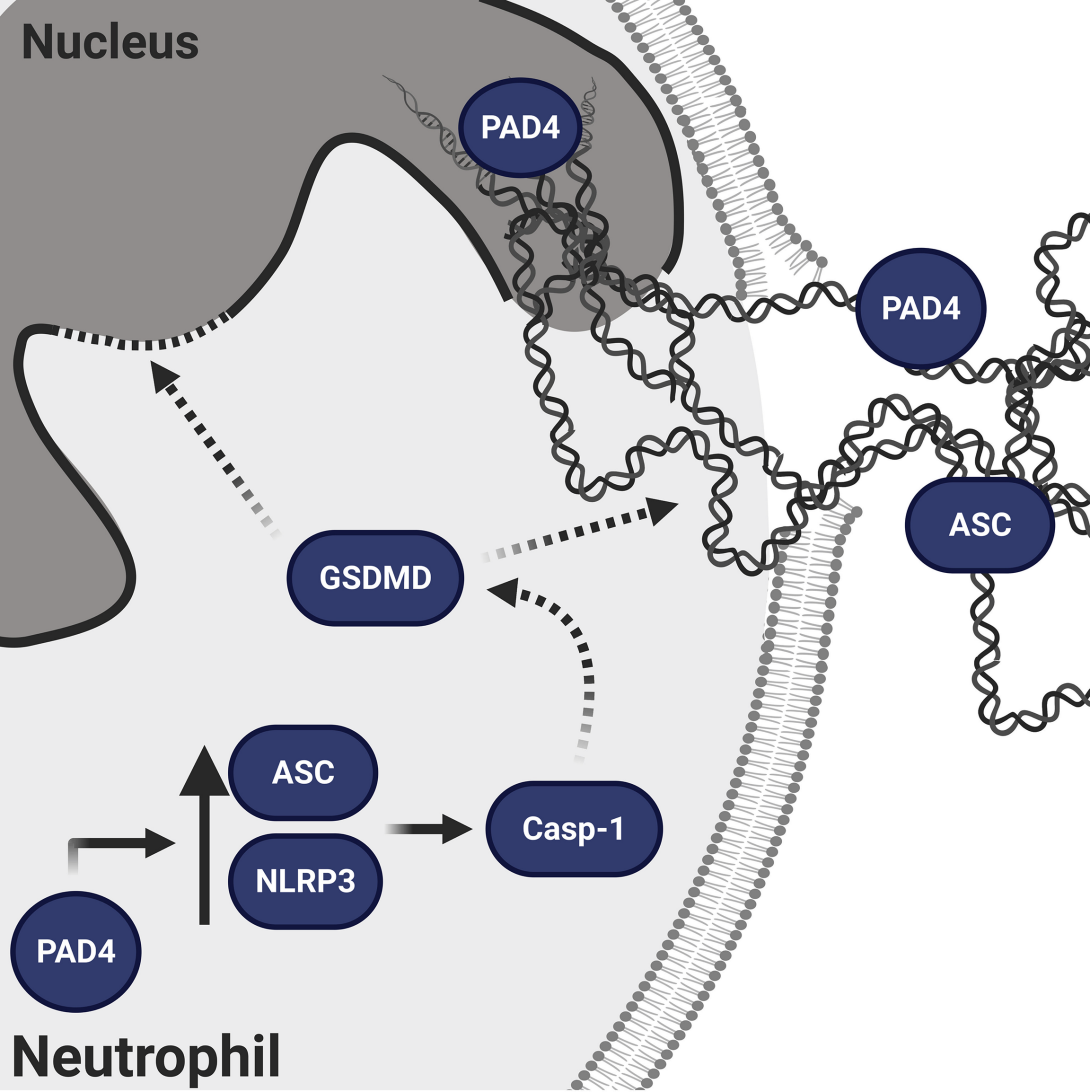
Schematic representation of the proposed mechanism of NLRP3/PAD4-induced NETosis in the absence of infection. PAD4 is present both in the nucleus and cytoplasm (15). In the nucleus, PAD4 orchestrates chromatin decondensation, whereas in the cytoplasm, PAD4 increases NLRP3 and ASC protein levels post-transcriptionally, thus favoring NLRP3 inflammasome/ASC speck assembly. The NLRP3 inflammasome activates caspase-1, which is known to generate the N-terminal fragment of GSDMD pore that facilitates nuclear expansion (49) and nuclear permeabilization (50). In addition to GSDMD, caspase-1 has many other intracellular substrates (54) and therefore its activation could support the cytoskeletal and nuclear disassembly necessary for NETosis (12).

In 2018, two groups of investigators clearly demonstrated that GSDMD pore formation is implicated in NETosis in an NLRP3 inflammasome–independent manner. They proposed that GSDMD is activated either by neutrophil proteases that play a role in NETosis and can cleave GSDMD to its active fragments (49) or by caspase-11-mediated GSDMD cleavage after cytosolic infection by gram-negative bacteria (50). However, as our experiments were exclusively performed under sterile conditions, the observed effects are most likely mediated by caspase-1-dependent mechanisms. In macrophages the cleaved N-terminal GSDMD fragment is established as pore-forming compound (51), while in neutrophils, the elastase (NE)-dependent activation of GSDMD can lead to alternative GSDMD cleavage and localization resulting in pyroptosis-independent signaling (52). Nevertheless, caspase-11 as well as NE-dependent GSDMD processing affect nuclear extension (49) and nuclear permeabilization (50) in neutrophils. In this context, the importance of caspase-1 or 11 in NETosis is consistent with our collaborative observations showing that caspase-1/11-deficient mice do not expel NETs (53) and that pharmacological perturbation of caspase-1 activity using a specific caspase-1 inhibitor resulted in impaired NET formation (**Figure S5**). Interestingly, all types of sterile stimulation that we tested produced significant ASC speck/inflammasome assembly (**Figure 1**). However, further research is needed to clarify the exact roles of caspase-1 and GSDMD in neutrophil activation. Since occasional speck formation was observed in resting neutrophils and not in naive mononuclear cells, it appears that neutrophils are already primed for inflammasome formation (**Figure 6**).

Apart from NLRP3, other inflammasomes present in neutrophils, such as the NLRC4, NLRP1 and AIM2 inflammasomes, are also known to induce ASC speck-dependent caspase-1 activation (19, 26, 55). NLRP3 and NLRP1 expression levels in neutrophils are higher than in macrophages (56) and especially NLRP1 was recently described as mediator of anthrax lethal toxin induced neutrophil activation (55). Additionally, the AIM2 inflammasome is vastly activated by cytosolic dsDNA, which is a characteristic of NETosis. These observations by others may explain why residual ASC speck formation is still observed in *Nlrp3^–/–^* (**Figure 1E**) or MCC950-treated neutrophils (**Figures 4B, C**). Further investigations are needed to determine the role of inflammasomes other than NLRP3 in NETosis.

PAD4-dependent citrullination events are important in NETosis (12, 15). PAD4 deficiency protects mice from thrombosis, ischemia reperfusion injury, and age-related tissue fibrosis (5, 15). The observation that NLRP3 is linked to similar conditions and functional decline in aging (57, 58) suggested to us that PAD4 could also be regulating the NLRP3 inflammasome. Indeed, we found an impaired ASC speck formation in neutrophils from PAD4-deficient mice when compared with wild-type neutrophils (**Figure 2A**). Recently, a study was published showing that PAD enzymes were also necessary for NLRP3/ASC speck formation in macrophages. While Cl-amidine treatment fully inhibited ASC speck formation, PAD4 deficiency alone, however, had no effect on ASC speck formation (44) (**Figure S3**), showing that PAD4 is more important in neutrophil inflammasome assembly than in macrophages. These observations point to a compensatory effect of other PAD enzymes in macrophages, such as PAD2, which is the main PAD isoform in these leukocytes (59). Since post-translational modifications such as phosphorylation, ubiquitination, or the change in the charge of a single amino acid are known to modify the oligomerization or stability abilities of the NLRP3 inflammasome (60, 61), citrullination of arginine residues by PAD4 could regulate NLRP3 inflammasome oligomerization. Particularly, treatment with the calcium ionophore ionomycin increases intracellular calcium levels and thus likely induce the enzymatic activity of PAD4 with subsequent citrullination of inflammasome components. The same mechanisms is assumed for the potassium ionophore nigericin, by indirectly increasing cytosolic calcium concentrations with subsequent inflammasome activation (62). Another way by which PAD4 could promote inflammasome assembly is the upregulation of NLRP3 production (26, 43). PAD4-dependent citrullination of the NFκB subunit p65 has been described as a mediator of its nuclear translocation (63). Thus, PAD4 elevation in disease (15) could regulate NLRP3 and ASC protein levels in neutrophils and, when overexpressed, also in iBMDMs, as we observed in our study (**Figures 2B**, **3C**). Although nigericin rarely induces caspase-1 activation in unprimed macrophages and monocytes (64, 65), PAD4 overexpression results in LPS-independent inflammasome activation upon stimulation with nigericin (**Figures 3A, B**) without affecting IL-1ß mRNA levels (**Figure S4C**). Remarkably, relative mRNA levels of ASC or NLRP3 were not different in wild-type or PAD4-deficient neutrophils as well as in empty vector or PAD4 overexpressing iBMDMs (**Figures 2C**, **3D**). The post-transcriptional effect of PAD4 on NLRP3 and ASC protein levels was further substantiated, as induction of transcription by LPS priming showed similar ASC and NLRP3 protein levels between wild-type and PAD4-deficient neutrophils (**Figure S2D**). Consequently, these findings show that the inflammasome protein levels are most likely regulated by an increase in translation or a reduced clearance of NLRP3. Indeed, it will be interesting to investigate the mechanism by which citrullination controls NLRP3 inflammasome in the future.

We examined how NLRP3 deficiency impaired NETosis by using time-lapse microscopy. This revealed that initiation of NETosis appeared normal, including plasma membrane vesiculation and nuclear rounding (45); however, following rounding there was diminished rupture of the nuclear envelope and, importantly, almost absent plasma membrane rupture (**Figures 4D, F**). While GSDMD was shown to induce nuclear membrane permeability in neutrophils, it has been suggested that this could promote nuclear membrane breakdown (50). However, plasma membrane rupture during pyroptotic cell death is a well-established event of GSDMD activity after inflammasome activation (51). Since we observed that plasma membrane permeabilization occurs prior to plasma membrane rupture in NETosis (12), inflammasome-dependent GSDMD pore likely prepares the plasma membrane for rupture. In neutrophils, subjected to sterile stimulation, this process is NLRP3 dependent.

Our data point to an important connection between inflammasome/ASC speck and NETosis (**Figure 6**) and thereby possibly promoting inflammatory noninfectious disorders. Diseases that are associated with increased PAD4 expression in neutrophils and elevated susceptibility to NETosis could possibly be linked to elevated NLRP3 inflammasome assembly in both neutrophils and macrophages, as we observed. Neutrophils from diabetic patients for instance showed a four-fold increase in PAD4 levels leading to augmented NETosis (7), and NLRP3 inflammasome is long well-known in type 2 diabetes (66). PAD4-dependent NETosis is also a crucial part of venous thrombosis (14). Thus it is not surprising that we observed that NLRP3 deficiency also diminished venous thrombosis (**Figure 5**). Moreover, considering the clinical importance of excessive IL-1ß generation in a wide variety of thrombo-inflammatory disorders, as shown by the CANTOS trial (67, 68), PAD-dependent regulation of NLRP3 protein levels could be an important mechanism in inflammasome-driven diseases and makes PADs promising new targets in the therapy of cardiovascular diseases (15, 69, 70).

It is likely that inflammasome-driven chronic diseases could be promoted further by neutrophilic inflammasome, as we found ASC speck expelled with extracellular chromatin on NETs (**Figure 1C**). These ASC specks could be taken up by other cells and propagate inflammation. Indeed, ASC has been described as a “prionoid”, that, when phagocytosed by macrophages, induce inflammasome assembly (27, 71, 72).

To conclude, here we have evaluated the link between canonical inflammasome and NETosis in sterile environment (**Figure 6**). Our results show inflammasome-dependent signaling is part of NETosis and PAD4 regulates ASC speck formation. These observations open many new avenues that may provide a base for new approaches in the prevention and treatment of inflammatory diseases. Finally, this work revealed additional positive effects of known drugs in development. We show that an inhibitor of NLRP3 will also reduce the toxic effects of NETs and propose that PAD inhibitors may improve inflammasome-driven human disorders, including cardiovascular disease and thrombosis.

## Data Availability Statement

The original contributions presented in the study are included in the article/**Supplementary Material**. Further inquiries can be directed to the corresponding author.

## Ethics Statement

The studies involving human participants were reviewed and approved by Office of Clinical Investigations at Boston Children’s Hospital. The patients/participants provided their written informed consent to participate in this study. The animal study was reviewed and approved by Institutional Animal Care and Use Committee of Boston Children’s Hospital or the Regierungspräsidium Tübingen.

## Author Contributions

PM, RN, SF, LM, KA, LC, DC, SG, NS, LS, VM, and ANRW performed experiments and analyzed the data. ANRW provided reagents. PM, RN, RES, CMW, HW, and DDW designed the research. PM and DDW designed the project and wrote the manuscript. All authors contributed to the article and approved the submitted version.

## Funding

This work was supported by a grant from National Heart, Lung, and Blood Institute of the National Institutes of Health (grant R35 HL135765) and a Steven Berzin family support to DDW, an Individual Erwin Deutsch fellowship by the German, Austrian and Swiss Society of Thrombosis and Hemostasis Research to RES, a Whitman fellowship (MBL) to DDW, and an Individual Marie Skłodowska-Curie Actions fellowship by the European Commission (796365 - COAGULANT) to PM. ANRW was funded by the Deutsche Forschungsgemeinschaft (TRR156/2 –246807620) and a research grant (We-4195/15-19). CMW was supported by the Division of Intramural Research, NHLBI, NIH.

## Conflict of Interest

DDW is on the Scientific Advisory Board of Neutrolis, a preclinical-stage biotech company focused on DNases.

The remaining authors declare that the research was conducted in the absence of any commercial or financial relationships that could be construed as a potential conflict of interest.
