# Supplementary material for: NLRP3 Inflammasome Assembly in Neutrophils Is Supported by PAD4 and Promotes NETosis Under Sterile Conditions
Source: Front Immunol. 2021 May 28;12:683803. doi: 10.3389/fimmu.2021.683803 (PMC8195330; doi:10.3389/fimmu.2021.683803)
Supplement: Supplementary file 1 [file DataSheet_1.pdf]

SUPPLEMENTARY MATERIAL AND METHODS

Isolation and culture of mouse primary bone marrow–derived macrophages

Bone marrow–cells were harvested by flushing the mouse femur and tibia. Macrophage development was promoted for 7 days in DMEM supplemented with 10% heat-inactivated FBS, 1% penicillin/streptomycin, and macrophage stimulating factor (M-CSF, 20 ng/mL) on 35 mm glass-bottom dishes at a concentration of $2x10^6$ cells per dish. The culture medium was changed every second day. Where indicated, cells were pretreated with 200 µM Cl-amidine for 1 hour and primed afterwards for 4 hours with 1 µg/mL LPS, followed by stimulation with 10 µM nigericin for 30 minutes.

Western blot of PAD4

Western blot analysis were performed as described in the Material and Methods Section of the manuscript. In particular, membranes were stripped for for 20 minutes at RT using 0.5 M NaOH solution, blocked with 5% BSA TBS-T buffer, and incubated for 4 hours at RT using a custom-made mouse-specific PAD4 antibody (1:200, Thermo Fisher Scientific) directed against mouse PAD4 peptide DKEDPQASGMDFEDDKILD that does not cross react with mouse PAD2.

In vitro NET assay

In vitro NET assays were performed as described in the Material and Methods Section of the manuscript using $1.5x10^4$ human neutrophils in the absence (solv.control) or presence of 20 µM of the specific caspase-1 inhibitor AC-YVAD-cmk. Neutrophils were stimulated with vehicle control, nigericin (15 µM), MSU (0.2 mg/mL), or ionomycin (4 µM) for 4 hours.

SUPPLEMENTARY FIGURES

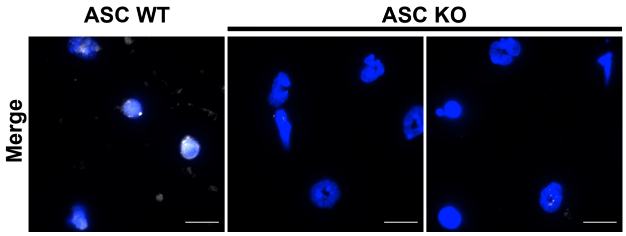

Supplementary Figure 1: Immunfluorescent validation of the used ASC antibody
Representative microscopy images of immunostainings using a mouse specific ASC antibody (Cell Signaling Technology; clone D2W8U) in unstimulated neutrophils from wild-type (ASC WT; left image) and ASC-deficient (ASC KO; right images) mice. Scale bar equals 10 µm.

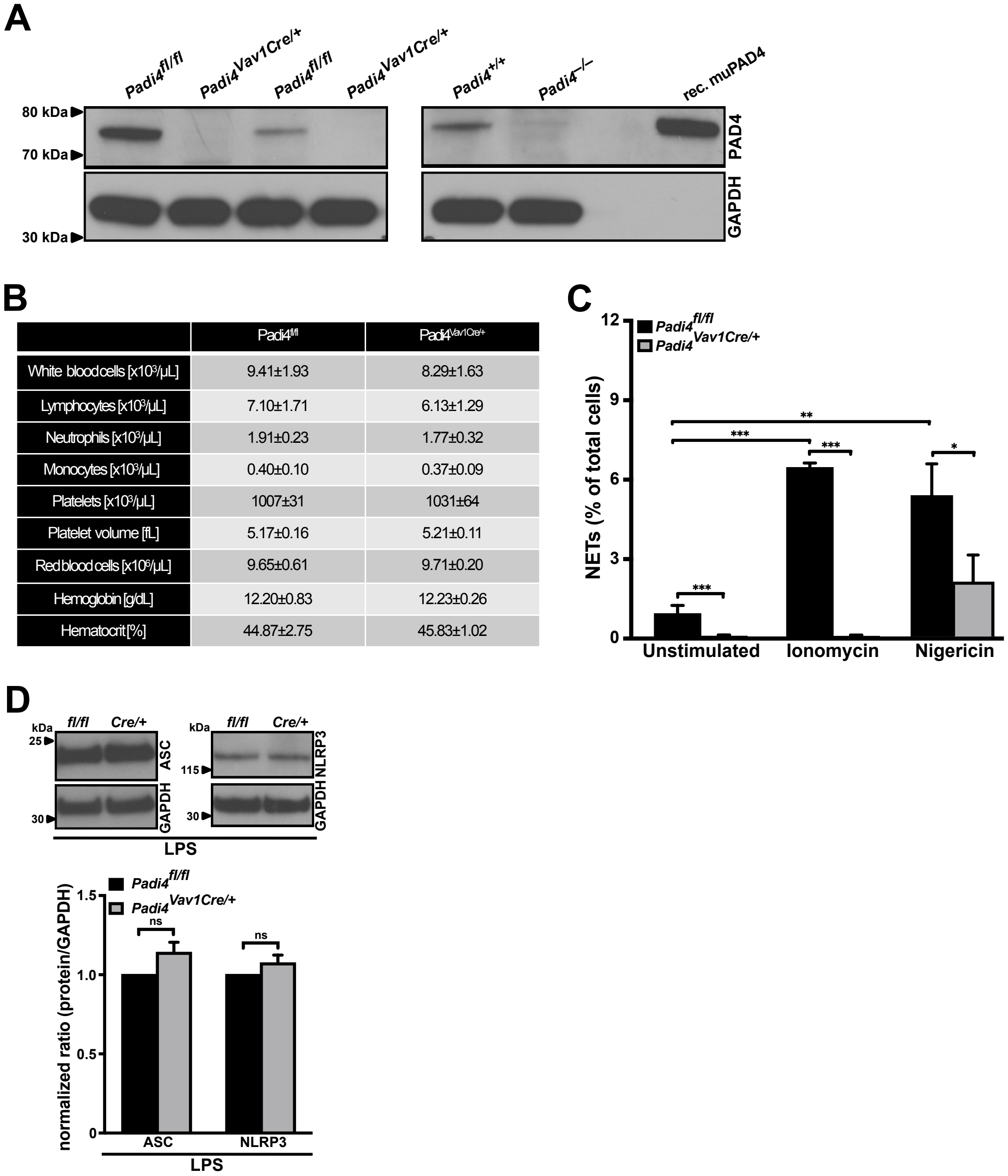

	$Padi4^{fl/fl}$	$Padi4^{Vav1Cre/+}$
White blood cells [x10³/µL]	9.41±1.93	8.29±1.63
Lymphocytes [x10³/µL]	7.10±1.71	6.13±1.29
Neutrophils [x10³/µL]	1.91±0.23	1.77±0.32
Monocytes [x10³/µL]	0.40±0.10	0.37±0.09
Platelets [x10³/µL]	1007±31	1031±64
Platelet volume [fL]	5.17±0.16	5.21±0.11
Red blood cells [x10⁶/µL]	9.65±0.61	9.71±0.20
Hemoglobin [g/dL]	12.20±0.83	12.23±0.26
Hematocrit [%]	44.87±2.75	45.83±1.02

Supplementary Figure 2: Characterization of hematopoietic cell-specific *Padi4*$^{Vav1Cre/+}$ mice and effect of LPS priming on ASC and NLRP3 protein levels

(**A**) Representative western blot of PAD4 protein expression of 4 experiments with neutrophils from wild-type (*Padi4*$^{fl/fl}$) and hematopoietic cell-specific Padi4-deficient mice (*Padi4*$^{Vav1Cre/+}$) on the left and with neutrophils from wild-type (*Padi4*$^{+/+}$) and global knockout mice (*Padi4*$^{-/-}$) on the right. rec. muPAD4; recombinant murine PAD4. (**B**) Arithmetic means ± SEM (n=6-7) of peripheral blood cell counts in male wild-type (*Padi4*$^{fl/fl}$) and hematopoietic cell-specific Padi4-deficient mice (*Padi4*$^{Vav1Cre/+}$). (**C**) Arithmetic means ± SEM (n=4) of NET formation by neutrophils from wild-type mice (*Padi4*$^{fl/fl}$, black bars) and hematopoietic cell-specific Padi4-deficient mice (*Padi4*$^{Vav1Cre/+}$, grey bars) in the absence (unstimulated) or presence of ionomycin

(4 μM) or nigericin (15 μM) for 4 hours. *$p<0.05$, **$p<0.01$ and ***$p<0.001$. (**D**) Representative western blots (upper panel) and arithmetic means ± SEM (lower panel, n=4 mice) of ASC and NLRP3 protein levels in naive neutrophils from wild-type ($Padi4^{fl/fl}$, black bars) or hematopoietic specific Padi4-deficient mice ($Padi4^{Vav1Cre/+}$, grey bars) in the presence of LPS (1 μg/mL) for 4 hours.

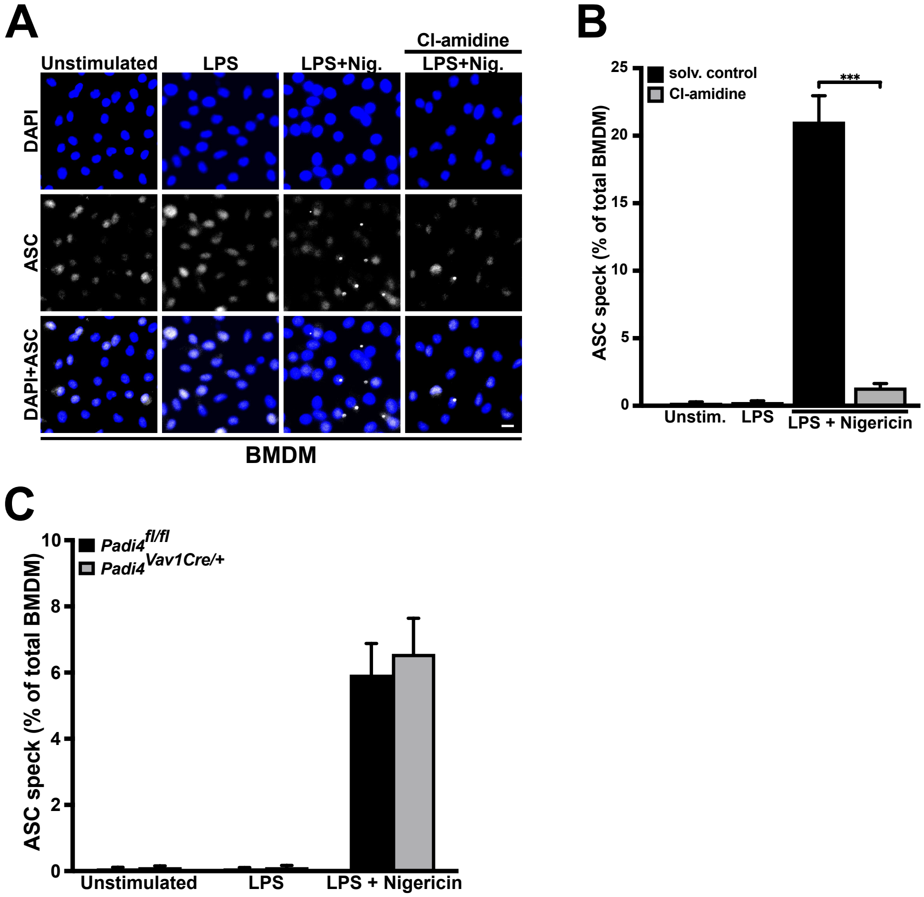

Supplementary Figure 3: ASC speck formation in primary bone marrow–derived macrophages is affected by PAD enzymes

(**A**) Confocal microscopy images of immunostained primary bone marrow–derived macrophages from wild-type mice in the absence (unstimulated) or presence of LPS (1 µg/mL, 4 hours), LPS and nigericin (10 µM, 0.5 hours), or LPS and nigericin upon 1 hour pretreatment with Cl-amidine (200 µM). Blue, DNA (DAPI); grey, ASC antibody staining. Scale bar equals 10 µm. Representative of n=8-12 experiments. (**B**) Arithmetic means ± SEM (n=8-12 mice) of percentage of ASC speck forming primary bone marrow–derived macrophages from wild-type mice in the absence (unstimulated) or presence of LPS (1 µg/mL, 4 hours), LPS and nigericin (10 µM, 0.5 hours), or LPS and nigericin upon 1 hour pretreatment with Cl-amidine (200 µM, grey bar). ***p<0.001 (**C**) Arithmetic means ± SEM (n=4-6 mice) of percentage of ASC speck formation in primary bone marrow–derived macrophages from wild-type ($Padi4^{fl/fl}$, black bars) and hematopoietic cell-specific Padi4-deficient mice ($Padi4^{Vav1Cre/+}$, grey bars) in the absence (unstimulated) or presence of LPS (1 µg/mL, 4 hours) or LPS and nigericin (10 µM, 0.5 hours).

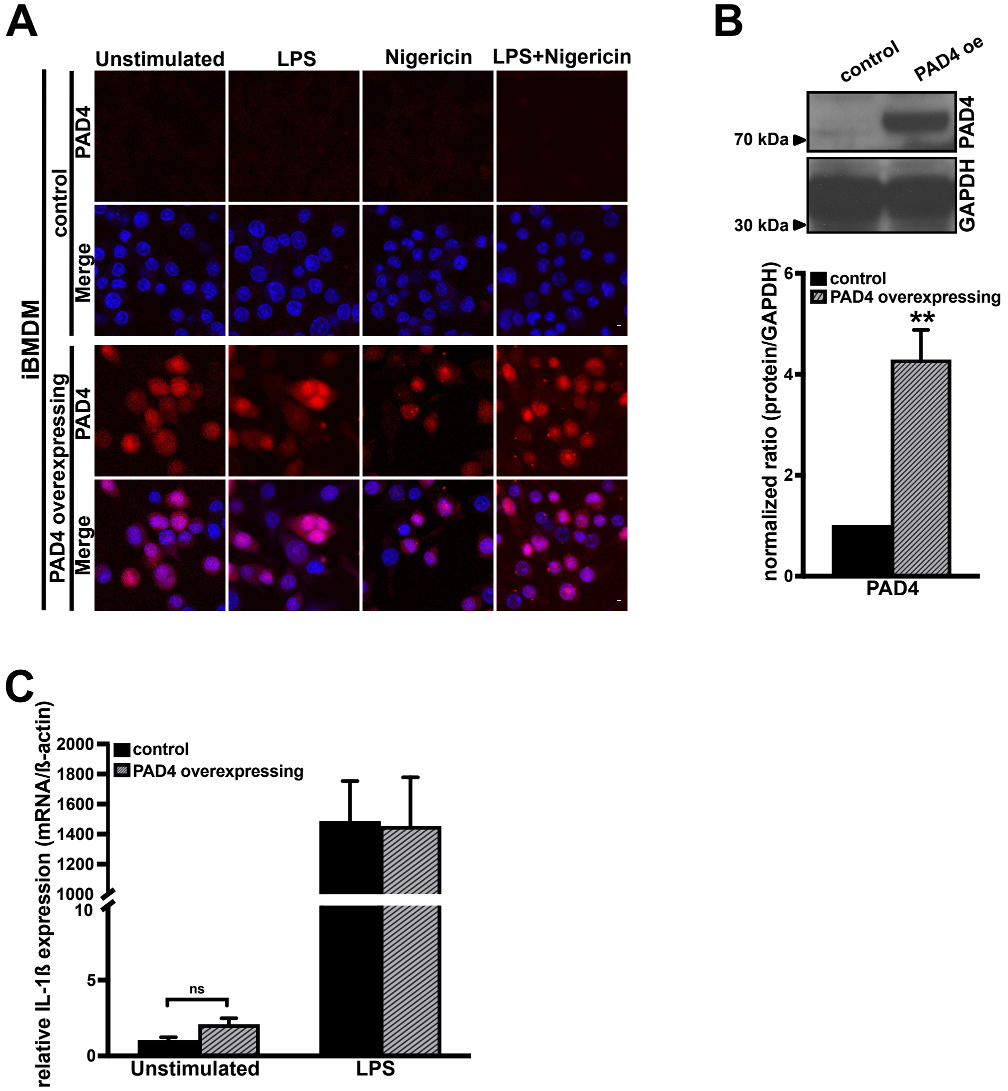

Supplementary Figure 4: PAD4 protein levels and IL-1ß mRNA level in naive or PAD4 overexpressing iBMDMs

(**A**) Confocal microscopy images of immunostained native mouse iBMDM (control, upper panel) and PAD4 overexpressing iBMDM (lower panel) in the absence (unstimulated) or presence of LPS (1 µg/mL), nigericin (15 µM), or LPS and nigericin (15 µM, 0.5 hours). Blue, DNA (Hoechst); red, PAD4 mScarlet. Scale bar equals 5 µm. Representative of n=4 experiments. (**B**) Representative western blot (upper panel) and arithmetic means ± SEM (lower panel, n=3) of PAD4 protein levels in unstimulated native iBMDM or PAD4 overexpressing (P4 oe) iBMDM. **p<0.01. (**C**) Arithmetic means ± SEM (n=4) of relative IL-1ß mRNA levels in unstimulated or LPS pretreated (4 hrs, 1 µg/ml) naive (control) or PAD4 overexpressing iBMDM.

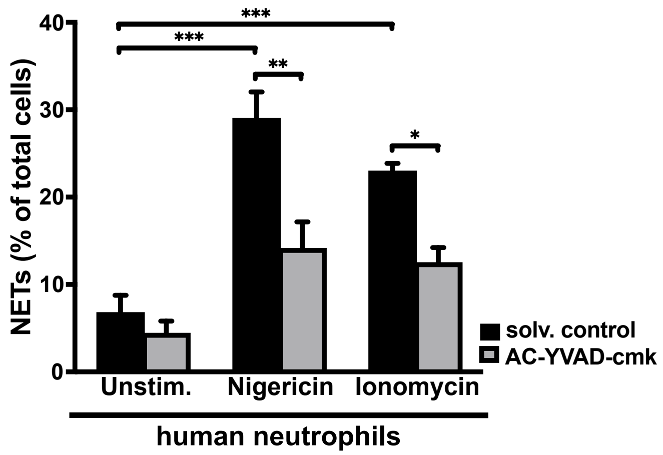

Supplementary Figure 5: Caspase-1 promotes activation-dependent NET formation

Arithmetic means ± SEM (right, n=5 donors) of NET formation in untreated (solvent control, black bars) or AC-YVAD-cmk pretreated (20 µM, grey bars) human neutrophils in the absence (unstimulated) or presence of nigericin (15 µM) or ionomycin (4 µM) for 4 hours. *$p<0.05$, **$p<0.01$ and ***$p<0.001$.

SUPPLEMENTARY MOVIES

Movie S1: Time-lapse visualization of NETosis by neutrophils from *Nlrp3*$^{+/+}$ mice

Representative time-lapse differential interference contrast (DIC, gray-scale) and spinning-disk confocal microscopy movie of NETosis over 4 hours by neutrophils from *Nlrp3*$^{+/+}$ mice in the presence of ionomycin. Blue, DNA (siR-DNA); red, endoplasmic reticulum and nuclear envelope (ER-tracker). Scale bar equals 10 µm.

Movie S2: Time-lapse visualization of NETosis by neutrophils from *Nlrp3*$^{-/-}$ mice

Representative time-lapse differential interference contrast (DIC, gray-scale) and spinning-disk confocal microscopy movie of NETosis over 4 hours by neutrophils from *Nlrp3*$^{-/-}$ mice in the presence of ionomycin. Blue, DNA (siR-DNA); red, endoplasmic reticulum and nuclear envelope (ER-tracker). Scale bar equals 10 µm.
