# Supplementary material for: NLRP3 Inflammasome Assembly in Neutrophils Is Supported by PAD4 and Promotes NETosis Under Sterile Conditions
Source: Front Immunol. 2021 May 28;12:683803. doi: 10.3389/fimmu.2021.683803 (PMC8195330; doi:10.3389/fimmu.2021.683803)
Supplement: Supplementary file 2 [file DataSheet_2.pdf]

Materials

Reagent or Resource	Resource	Identifier
Antibodies:		
anti mouse HRP conjugated	Bio-Rad	#1706516
anti mouse Alexa546	Thermo Fisher Scientific	#A10036
anti rabbit HRP conjugated	Sigma-Aldrich	#1706515
anti rabbit Alexa647	Thermo Fisher Scientific	#21244
ASC (mouse specific) (clone: D2W8U)	Cell Signaling Technology	#67824
ASC (clone: B-3)	Santa Cruz Biotechnology	#514414
caspase-1 (p-20) (clone: Casper-1)	AdipoGen	#AG-20B-0044-C100
GAPDH	Sigma-Aldrich	#G9545
goat anti mouse Alexa488	Thermo Fisher Scientific	#A10680
goat anti rabbit Alexa647	Thermo Fisher Scientific	#A21244
H4Cit	Millipore	#07-596
Ly6G (clone: 1A8)	BD Bioscience	#551459
NLRP3 (clone: Cryo-2)	AdipoGen	#AG-20B-0014-C100
NLRP3 (clone: D4D8T)	Cell Signaling Technology	#15101
PAD4	Thermo Fisher Scientific	custom made
Dyes:		
DAPI solution	Thermo Fisher Scientific	#62248
ER-tracker	Thermo Fisher Scientific	#E34250
Hoechst 33342	Invitrogen	#H3570
SiR-DNA	Cytoskeleton Inc.	#CY-SC007
Chemicals and compounds:		
AC-YVAD-cmk	InvivoGen	#inh-yvad
All-In-One RT MasterMix	Applied Biological Materials Inc.	#G485
Bradford reagent	Bio-Rad	#5000001
BSA	Sigma-Aldrich	#7906
Cl-amidine	Cayman Chemical	#10599
DMEM	Thermo Fisher Scientific	#10569-010
DMSO	Sigma-Aldrich	#D2650
ECL detection solution	Thermo Fisher Scientific	#32106
EDTA	Strem Chemicals	#93-1104
endotoxin-free BSA	Sigma-Aldrich	#9306
endotoxin-free mini-prep kit	Qiagen	#27104

Ethanol	Pharmco-Aaper	#111000200
FastAP	Fermentas	#E0651
FastDigest BsmBI	Fermentas	#FD0454
FBS	VWR	#97068-091
gRNA and primer	Integrated DNA Technologies	N/A
HEPES	Thermo Fisher Scientific	#15630-080
IL-1ß ELISA	BioLegend	#432601
ionomycin	Thermo Fisher Scientific	# I24222
isoflurane	Patterson Veterinary	# 07-893-2374
LDS buffer	Thermo Fisher Scientific	#B0007
LPS	InvivoGen	#tlrl-b5lps
MCC950	InvivoGen	#inh-mcc
M-CSF	ProSpec	#CYT-439
Mountant medium (DAPI)	Thermo Fisher Scientific	#P36966
nigericin	Sigma-Aldrich	#N7143
Padi4 cDNA	Origene	#MC220244
Paraformaldehyde	Electron Microscopy Sciences	#15710-S
PBS	Thermo Fisher Scientific	#14190-144
Penicillin/Streptomycin	Sigma-Aldrich	#P4333
Percoll	Sigma-Aldrich	#GE17-5445-01
phenol red-free RPMI 1640	Sigma-Aldrich	#R7509
Phosphatase inhibitor	Thermo Fisher Scientific	#78420
PMA	Sigma-Aldrich	#P1585
polybrene	Santa Cruz Biotechnology	#sc-134220
Protease inhibitor	Thermo Fisher Scientific	#87786
PureLink™ RNA Mini Kit	Thermo Fisher Scientific	#12183018A
reducing agent	Thermo Fisher Scientific	#B0009
RIPA buffer	Thermo Fisher Scientific	#89901
RPMI 1640 (L-Glutamine)	Thermo Fisher Scientific	#11875-093
SYBR Green SuperMix	Quanta Biosciences	#95054-100
T7 ligase	Enzymatics	#L602L
Tissue-Tek® O.C.T.™	VWR	#4583
Triton-X 100	Sigma-Aldrich	#93426
Tween 20	Sigma-Aldrich	#P1379
Materials:		
5-0 nylon suture	Ethicon	#G695G
6-0 monocryl suture	Ethicon	#Y492G
7-0 polypropylene suture	Ethicon	#8696G
0.45µm filter	Pall Corporation	#4614

40 µm cell strainer	Sigma-Aldrich	#431750
35 mm glass bottom petri dish	MatTek	#P35G-1.5-20-C
24-well glass bottom plate	CellVis	#P24-1.5H-N
4-12% Bis-Tris gel	Thermo Fisher Scientific	#NW04122BOX
6-well plate	VWR	#734-2323
96-well glass bottom plate	Greiner bio-one	#655892
coverslips	Sigma-Aldrich	#CLS285022
EDTA coated vacutainer	Becton Dickinson	#366643
microscopy slides	Fisher Scientific	#12-550-123
PVDF membrane	Thermo Fisher Scientific	#IB401001
Devices:		
FACSARIA II Cell Sorter	BD Bioscience	N/A
LSRFortessa flow cytometer	BD Bioscience	N/A
Hemavet	Drew Scientific	N/A
iBlot	Thermo Fisher Scientific	N/A
StepOnePlus RealTime PCR System	Applied Biosystems	N/A
Software:		
AxioVision Version	Carl Zeiss Microscopy	N/A
Elements Version	Nikon	N/A
Fiji / ImageJ Version 2.0.0	Schindelin, J.; Arganda-Carreras, I. & Frise, E. et al. (2012)	PMID 22743772
GraphPad Prism (Version 8)	GraphPad Software	N/A
MS Excel	Microsoft	N/A
Olympus FluoView viewer software Version 3.0	Olympus	N/A
StepOne Software v2.1	Thermo Fisher Scientific	N/A
